# The Composition of the HDL Particle and Its Capacity to Remove Cellular Cholesterol Are Associated with a Reduced Risk of Developing Active Inflammatory Rheumatoid Arthritis

**DOI:** 10.3390/ijms252010980

**Published:** 2024-10-12

**Authors:** Marcia Benacchio Giacaglia, Vitoria Pires Felix, Monique de Fatima Mello Santana, Leonardo Szalos Amendola, Perola Goberstein Lerner, Sibelle D. Elia Fernandes, Cleber Pinto Camacho, Marisa Passarelli

**Affiliations:** 1 Programa de Pós-Graduação em Medicina, Universidade Nove de Julho (UNINOVE), Sao Paulo 01525-000, Brazil; marciabenacchio@uni9.edu.br (M.B.G.);; 2 Laboratório de Lípides (LIM10), Hospital das Clínicas da Faculdade de Medicina da Universidade de Sao Paulo (HCFMUSP), Sao Paulo 01246-000, Brazil; 3 Departamento de Reumatologia, Hospital do Servidor Público Municipal (HSPM), Sao Paulo 01532-000, Brazil; 4 Laboratório de Análise Clínicas, Hospital do Servidor Público Municipal (HSPM), Sao Paulo 01532-000, Brazil

**Keywords:** rheumatoid arthritis, inflammation, atherosclerosis, HDL, cholesterol efflux

## Abstract

In rheumatoid arthritis (RA), the risk of cardiovascular death is 50% higher compared to the general population. This increased risk is partly due to the systemic inflammation characteristic of RA and changes in the lipoprotein profiles. This study investigated plasma lipid levels, lipid ratios, and the composition and functionality of high-density lipoprotein (HDL) in control individuals and RA subjects based on the disease’s inflammatory score (DAS28). This study included 50 control (CTR) individuals and 56 subjects with RA, divided into remission/low-activity disease (DAS28 < 3.2; *n* = 13) and active disease (DAS28 ≥ 3.2; *n* = 43). Plasma lipids (total cholesterol, TC; triglycerides, TG) and the HDL composition (TC; TG; phospholipids, PL) were determined using enzymatic methods; apolipoprotein B (apoB) and apoA-1 were measured by immunoturbidimetry. HDL-mediated cholesterol efflux and anti-inflammatory activity were assessed in bone marrow-derived macrophages. Comparisons were made using the Mann–Whitney test, and binary logistic regression was used to identify the predictors of active RA. A *p*-value < 0.05 was considered significant. TC, HDLc, and the TC/apoB ratio were higher in RA subjects compared to the CTR group. Subjects with active disease exhibited higher levels of TG and the TG/HDLc ratio and lower levels of HDLc, the TG/apoB ratio, TC, and apoA-1 in HDL particles compared to those with remission/low-activity RA. Increased levels of HDLc [odds ratio (OR) 0.931, 95% CI = 0.882–0.984], TC/apoB (OR 0.314, 95% CI = 0.126–0.78), HDL content in TC (OR 0.912, 95% CI = 0.853–0.976), PL (OR 0.973, 95% CI = 0.947–1.000), and apoA-1 (OR 0.932, 95% CI = 0.882–0.985) were associated with a decreased risk of active disease, but BMI (OR 1.169, 95% CI = 1.004–1.360) and TG (OR 1.031, 95% CI = 1.005–1.057) were positively associated with active disease. A reduction in HDL-mediated cholesterol efflux increased the OR for active RA by 26.2%. The plasma levels of HDLc, along with the composition and functionality of HDL, influence the inflammatory score in RA and may affect the development of cardiovascular disease.

## 1. Introduction

Rheumatoid arthritis (RA) stands as one of the most prevalent autoimmune diseases, affecting up to 1% of the population in developed countries [1,2]. It exhibits a higher incidence in women, afflicting them two to three times more frequently than men, typically impacting individuals in their fourth to sixth decades of life [3]. Individuals with RA confront a 50% higher risk of death from cardiovascular disease (CVD) compared to the general population, primarily due to stroke and ischemic heart disease, with no apparent gender discrepancy [4]. The increase in cardiovascular (CV) risk in RA is attributed to several factors, particularly chronic inflammation, and alterations in the plasma lipid and lipoprotein profiles. Inflammation stands as a hallmark of RA, marked by alternating episodes of exacerbation and remission. The inflammatory and autoimmune profiles form the basis of metabolic alterations that play a pivotal role in the overall pathophysiological landscape of the disease.

The presence of dyslipidemia with elevations in low-density lipoprotein cholesterol (LDLc), associated with CV risk, is prevalent among individuals with RA [5]. Additionally, some studies conducted on RA subjects demonstrate a correlation between reduced CV risk and elevated HDL cholesterol (HDLc), mirroring the observations in the general population [6,7]. Conversely, a lipid paradox is also reported, where lower plasma levels of total cholesterol (TC) and LDLc are associated with increased CV risk in RA [8]. For instance, the coronary calcium score was four times higher in RA individuals with LDLc levels < 70 mg/dL than in controls without RA and similar levels of LDLc in plasma [9].

Plasma levels of HDLc are conventionally linked to CV protection across various populations, ages, and genders [10]. The inverse association between HDL and CVD is based on numerous antiatherogenic actions of this lipoprotein, encompassing the removal of excess cellular cholesterol and its transport to the liver for secretion into bile, ultimately facilitating its excretion in feces via reverse cholesterol transport. Additionally, HDL exhibits antioxidant, anti-inflammatory, and vasodilatory properties. Moreover, HDL promotes glucose homeostasis, modulates proteolysis, immune response, and hematopoiesis and carries numerous proteins, bioactive lipids, and microRNAs [11,12].

Nonetheless, the association between HDLc and improved CVD outcomes is not unequivocal in studies focusing on pharmacologically enhancing plasma HDLc or in Mendelian randomization studies. These findings underscore the role of HDL functionality in determining CV risk, which may not be adequately captured by measuring plasma HDLc or apolipoprotein A-1 (apoA-1), the primary protein component of HDL, as classical clinical metrics for HDL-mediated cardioprotection in various conditions.

In RA, dyslipidemia is linked to the intrinsic inflammatory state of the disease. Many studies have shown a connection between elevated disease activity indices, indicative of inflammation, and an increased risk of CVD [13]. Furthermore, there seems to be a correlation between the level of inflammatory activity and a decline in HDL function, primarily attributed to inflammatory cytokines binding to the surface of HDL, thus displacing apoA-1 from the particle [14,15]. The hypothesis that HDL functionality may be altered in RA and be linked to the inflammatory state of the disease was tested. The objective of this study was to assess the lipid profile of individuals with RA in comparison to controls and to investigate any potential association between the level of disease activity as measured by the Disease Activity Score with 28-Joint Counts (DAS-28) and plasma concentrations of HDLc, HDL particle composition, and functionality. Plasma lipids and HDL composition were similar between controls and RA subjects. Changes in HDL particle composition were observed when comparing subjects with remission/low-activity disease (DAS-28 < 3.2) to those with active disease (DAS-28 ≥ 3.2). Despite retaining anti-inflammatory capacity, HDL from individuals with active disease exhibited reduced efficacy in mediating cholesterol efflux from macrophages compared to those in remission/low-activity RA. Furthermore, the decreased levels of TC, PL, and apoA-1 in HDL particles, along with impaired cholesterol removal ability, heightened the odds ratio (OR) of developing active inflammatory RA.

## 2. Results

The control and RA groups were similar regarding age, sex, body mass index (BMI), and waist circumference (WC). The presence of obesity, dyslipidemia, statin use, menopausal status, use of hormone replacement therapy (HRT), use of hormonal contraceptives (HCs), and physical activity level (PAL) did not differ significantly between the groups. Most subjects with RA had moderate and active disease, according to the DAS28 index (Table 1).

In the RA group, 94.6% of subjects were on some disease-modifying antirheumatic drugs (DMARDs), and 14.3% were on corticosteroids (prednisone) as depicted in Table 2. Only two subjects (3.6%) were using corticosteroids at doses ≥ 7.5 mg/day, one taking 7.5 mg/day and the other 10 mg/day (Table 2).

Plasma levels of TC and HDLc, along with the TC/apoB ratio, were higher in the RA group. Additionally, the contents of PL and apoA-1 in the isolated HDL particles were elevated in RA (Table 3).

The division of the RA group into active disease (DAS28 ≥ 3.2) and remission/low-activity disease (DAS28 < 3.2) revealed that the age, sex, WC, and clinical characteristics were similar between the groups. Only BMI was significantly higher in the active disease group (Table 4).

In active disease compared to remission/low-activity RA, plasma levels of HDLc and TG, along with the TG/HDLc ratio, were higher, while the TC/apoB ratio was lower. Additionally, the HDL particles isolated from RA subjects with active disease exhibited reduced levels of TC and apoA-1 (Table 5).

**Figure 1:**
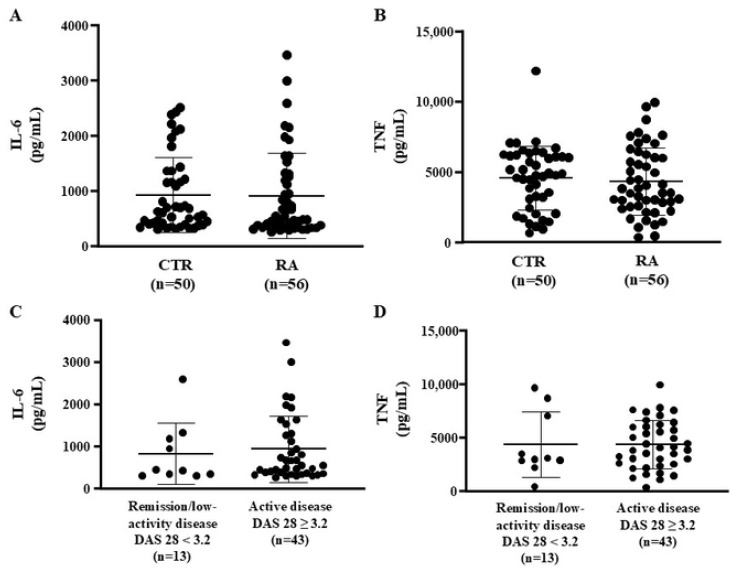
HDL anti-inflammatory activity. Bone marrow-derived macrophages were overloaded with acetylated LDL and exposed to HDL isolated from CTR (*n* = 50) and RA (*n* = 56) subjects. Following this, the cells were challenged with LPS, and the levels of IL-6 and TNF were determined by ELISA. Data were compared using the Mann–Whitney test between CTR and RA (**A**,**B**) or between RA cases with DAS 28 < 3.2 and DAS 28 ≥ 3.2 (**C**,**D**).

The anti-inflammatory action of HDL was analyzed in bone marrow-derived macrophages treated with HDL from the CTR and RA groups and challenged with lipopolysaccharide (LPS). The secretion of interleukin-6 (IL-6) and tumor necrosis factor (TNF) was similar between CTR and RA (Figure 1A,B) and did not change according to the disease activity (Figure 1C,D). A positive correlation (*r* = 0.381, *p* = 0.015) was observed between the IL-6 secretion and DAS28 score values in those with active disease.

**Figure 2:**
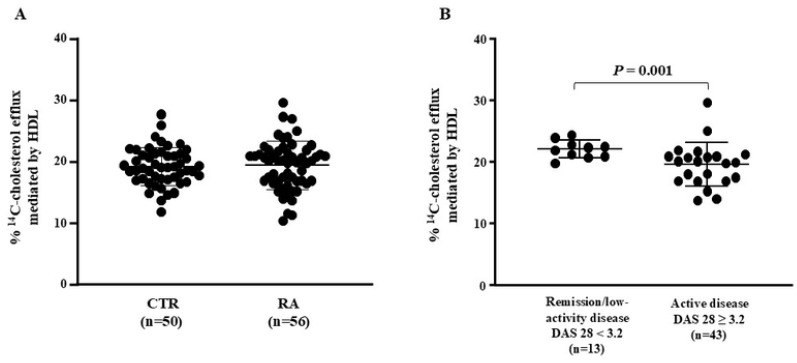
Cholesterol efflux mediated by HDL. Bone marrow-derived macrophages were overloaded with acetylated LDL and ^14^C-cholesterol and then exposed to HDL isolated from CTR (*n* = 50) and RA (*n* = 56) subjects as acceptors of cellular cholesterol. Data were compared using the Mann–Whitney test between CTR and RA (**A**) or between RA cases with DAS 28 < 3.2 and DAS 28 ≥ 3.2 (**B**).

Cholesterol efflux from macrophages was similar between HDL from the CTR and RA groups (Figure 2A). However, it was reduced by 16.76% in active disease compared to remission/low-activity RA (Figure 2B), with a statistical power of 0.82.

The significant predictors for RA with active disease are shown in Table 6, as revealed by the binary logistic regression model. The concentrations of HDLc [x^2^_(1)_ = 8.223; *p* = 0.011, Cox–Snell R^2^ = 0.137; (OR = 0.931; 95% CI = 0.882–0.984)], TC/apoB ratio [x^2^_(1)_ = 8.382; *p* = 0.013, Cox–Snell R^2^ = 0.139; (OR = 0.314; 95% CI = 0.126–0.781)], contents of HDL in TC [x^2^_(1)_ = 11.153; *p* < 0.007, Cox–Snell R^2^ = 0.181; (OR = 0.912; 95% CI = 0.853–0.976)], PL [x^2^_(1)_ = 4.310; *p* = 0.048, Cox–Snell R^2^ = 0.074; (OR = 0.973; 95% CI = 0.947–1.000)], apoA-1 [x^2^_(1)_ = 7.610; *p* = 0.013, Cox–Snell R^2^ = 0.127; (OR = 0.932; 95% CI = 0.882–0.985)], and HDL-mediated cholesterol efflux [x^2^_(1)_ = 10.088; *p* = 0.007, Cox–Snell R^2^ = 0.165; (OR = 0.738; 95% CI = 0.591–0.921)] were negative predictors. On the other hand, BMI [x^2^_(1)_ = 4.531; *p* = 0.044, Cox–Snell R^2^ = 0.078; (OR = 1.169; 95% CI = 1.004–1.360)] and plasma levels of TG [x^2^_(1)_ = 8.242; *p* = 0.020, Cox–Snell R^2^ = 0.137; (OR = 1.031; 95% CI = 1.005–1.057)] were positive predictors of active disease.

## 3. Discussion

Cardiovascular disease is prevalent in RA, and while controlling traditional CV risk factors and clinically managing the disease may not fully mitigate the risk of CV morbidity and mortality, non-traditional risk factors such as oxidative stress and alterations in the lipoprotein profile and function contribute to residual risk. The findings of this study emphasize the potential importance of HDL composition and functionality, particularly in removing excess cellular cholesterol, in predicting the risk of active RA. Considering the inflammatory nature of RA, this may impact the development of atherosclerosis in subjects with more active disease.

In the present study, the higher concentrations of HDLc observed in the RA group compared to the CTR group may be because the majority of RA individuals (94.6%) were on DMARDs. Seventy-one percent of RA subjects were using methotrexate, which, by reducing the inflammatory process, could lead to an elevation in HDLc levels. Georgiadis et al. [16] evaluated HDLc in 58 subjects with active RA without previous treatment and after 12 months of treatment with methotrexate and prednisone (7.5 mg/day), compared to 63 healthy controls. It was observed that the HDLc in the pre-treatment RA group was significantly lower than in the control group, while in the post-treatment period, the HDLc concentration was higher compared to pre-treatment and controls. The elevation of HDLc contributed to the higher observed concentration of TC in the RA group, consequently leading to a higher TC/apoB ratio, in the presence of similar levels of apoB in both groups.

Plasma levels of TG were higher in the active RA group compared to the RA group in remission/low-activity disease, which is consistent with previous findings [17,18]. The release of IL-6 and TNF results in the negative regulation of the genes encoding lipoprotein lipase and hepatic lipase, reducing the clearance of TG-rich particles. TG levels were positively associated with small dense LDLs in individuals with active RA [19]. Moreover, the TG/HDLc and TC/apoB ratios, indicative of the formation of small dense LDLs, were higher and lower, respectively, in the active RA group compared to the remission/low-activity disease group.

The current work evidenced that individuals with active RA had lower concentrations of HDLc compared to those with disease in remission/low-activity disease. A study compared 56 participants with active RA with 56 in disease remission and found significantly lower HDLc levels in those with active disease [20]. Inflammation reduces the synthesis of apoA-1 and decreases the half-life of HDL in plasma, contributing to lower HDLc concentrations in individuals with active RA. HDL exhibits an increase in the serum amyloid A (SAA) content and a decrease in the apoA-1 content during the acute-phase response. Additionally, there is a decrease in lecithin cholesterol acyltransferase (LCAT), cholesteryl ester transfer protein (CETP), hepatic lipase (HL), and paraoxonase 1 (PON1). Secretory phospholipase A2 (sPLA2) is activated by SAA, which displaces apoA-1 from HDL particles and increases HDL catabolism, contributing to reduced HDLc concentrations. The reduced expression of CETP observed during the acute-phase response is linked to decreased retinoid X receptor (RXR)/liver X receptor (LXR) activity in the liver [21]. The levels of sPLA2 were associated with the presence of subclinical atherosclerotic disease and the severity of disease in individuals with early RA [22]. Overexpression of endothelial lipase (EL), which has phospholipase A-1 activity, reduces HDLc levels, whereas inhibition of EL increases HDLc levels. The treatment of cultured endothelial cells with TNF or IL-1 beta has been shown to overexpress EL, reducing HDLc levels. If similar outcomes take place in vivo, this also may explain the lower HDLc levels disclosed in active RA subjects in the present study [17,23].

The lower concentration of HDLc is generally a consequence of autoimmune disease and inflammatory activity. However, some studies support the possibility that HDL could be involved in triggering autoimmune disease by regulating the proliferation of hematopoietic stem cells in the bone marrow [24]. Additionally, HDL can directly modulate immune cell function in the later stages of the immune response. Furthermore, changes in the concentration and function of HDL lead to the development of autoimmune phenotypes in animal models. In the Copenhagen General Population Study, individuals with reduced HDLc (<39 mg/dL) had a hazard ratio of 1.51 (95% CI: 0.94–2.42) for developing RA compared to individuals with high HDLc (≥77 mg/dL) [25].

In a study conducted by McMahon et al., which involved 154 women with SLE, 48 women with RA, and 72 healthy control women, a higher concentration of pro-inflammatory HDL was found in the RA group compared to the healthy control group. Similarly to the present study, their work showed significantly higher levels of HDLc in women with RA compared to healthy women, and 52.1% of the RA subjects used methotrexate [26].

Disease activity brings many undesirable consequences to the HDL particle. Many studies already advocated that an inflammatory state might be able to impair HDL’s major atheroprotective effect [27]. The usual characteristics of HDL are compromised, including its role in the reverse cholesterol transport pathway, with a worsening of HDL’s cholesterol efflux capacity (CEC), as well as an increase in oxidized PL in LDL and the induction of monocyte migration, ultimately reducing the atheroprotective functions and increasing the risk of CVD [28]. In the context of inflammation, the expression and activity of hepatic PON1 decrease, potentially contributing to the increased oxidation of LDL [14]. Pro-inflammatory cytokines decrease apoA-1 synthesis and the expression of ATP-binding cassette transporter A-1 (ABCA-1), ATP-binding cassette transporter G-1 (ABCG-1), scavenger receptor class B type 1 (SR-B1), and apolipoprotein E in macrophages, leading to a lower efflux of cholesterol to HDL. The expression of SR-B1 is regulated by peroxisome proliferator-activated receptors (PPARs) and the farnesoid X receptor (FXR). Consequently, the reduction in PPAR/RXR and FXR/RXR activities in the liver may mediate the decline in SR-B1 during the acute-phase response, leading to decreased delivery of cholesteryl esters to hepatocytes [29,30,31]. The structurally modified HDL by inflammation is a weak acceptor of cellular cholesterol, and it may even transfer cholesterol back to macrophages. Inflammatory cytokines decrease the synthesis and activity of LCAT, diminishing the generation of esterified cholesterol, which reduces both the production of normal spherical HDL and the capacity of HDL to transport cholesterol to the liver and steroidogenic organs [28].

The concentrations of TC and apoA-1 in HDL particles were lower in subjects with active RA compared to those with disease in remission/low disease activity, which may have impacted the ability of HDL to mediate macrophage cholesterol efflux. Inflammation not only decreases HDLc levels, but it also reduces the EC and apoA-1 content of HDL particles [32].

The capacity of apoB-depleted serum, consisting solely of HDL as a lipoprotein component, to mediate CEC from macrophages has been directly associated with CV protection in several clinical trials, regardless of the plasma HDLc concentration [33]. Ronda et al. [34] observed lower CEC mediated by serum from individuals with RA compared to controls. In addition, the CEC was impaired in individuals with RA with high disease activity in comparison to those with low disease activity. The reduction in CEC was attributed to the ABCG-1-mediated pathway, with an inverse association with disease activity, highlighting the impact of inflammation and autoimmunity on HDL function. There was no difference in the serum-mediated efflux through SR-B1, ABCA-1, or aqueous diffusion in subjects with RA compared to controls [34].

The CEC mediated by ABCA-1 was associated with a lower number and progression of atherogenic plaques in individuals with a low C-reactive protein (CRP) concentration, non-users of prednisone, and those using biological DMARDs at the initial assessment. Interestingly, the CEC was associated with the rapid progression of atherosclerosis in those with a high CRP concentration at the initial assessment, users of prednisone, and non-users of methotrexate or biological DMARDs. This suggests that the ABCA-1-mediated CEC contributes to a pro-atherogenic state when RA is not under inflammatory control [35].

Weber et al. did not find changes in CEC in subjects with RA divided into two groups: one with an increase and the other with a decrease in hs-CRP ≥ 10 mg/L in two consecutive annual visits. However, as mentioned by the authors, the study had limitations since heterogeneous reasons contributed to the increased inflammation besides RA, including the concomitant presence of infections, trauma, and other conditions [36]. A previous study from the same group showed that a reduction in inflammation was associated with a significant improvement in HDL cholesterol efflux capacity [37].

In a systematic review and meta-analysis comprising 11 selected studies (six observational and five interventional), the CEC was determined either by serum depleted of apoB (in most studies) or by HDL isolated from individuals with RA. There was no difference in the CEC despite the reduction in HDLc concentration in those with RA compared to healthy controls. In two interventional studies, the CEC in individuals with RA was improved despite no variation in the HDLc concentration after treatment with DMARDs [38].

In the present investigation, the intrinsic HDL ability to remove cell cholesterol was similar when comparing the CTR group with the RA group including all levels of disease activity. On the other hand, by dividing subjects with RA according to the inflammatory stage of the disease, a 16.76% reduction was observed in cholesterol efflux from macrophages mediated by HDL isolated from subjects with active RA in comparison to HDL from subjects with the disease in remission/low-activity. This finding agrees with the concept that inflammation impacts HDL function and is related to a more pronounced CV risk. Charles-Schoeman et al. [39] found similar results, reporting no difference in cholesterol efflux between RA patients and healthy control subjects. However, it is important to note that, unlike in the present investigation, the authors employed a different method to obtain HDL, and they did not address the composition of the HDL particles or their role in predicting disease activity.

In inflammation, HDL oxidation exerts pro-inflammatory effects on monocytes by promoting the upregulation of platelet-derived growth factor receptor beta, thereby increasing their chemotaxis and TNF release. Monocytes from individuals with low HDLc levels exhibited increased expression of inflammatory genes, including TNF [40]. Moreover, monocytes can differentiate into M1 classically activated macrophages promoting inflammation and M2 macrophages involved in the resolution of inflammation. Humoral factors can alter the balance between M1 and M2 macrophages; and in mice, HDL increased the expression of M2 macrophage markers, leading to significant changes in the content and characteristics of monocyte-derived macrophages, as well as the regression of atherosclerotic plaques [41].

The analysis of HDL’s role in mitigating endothelial dysfunction, as indicated by IL-6 production, showed lower effectiveness in individuals with low plasma HDLc concentrations compared to those with medium or high levels [42]. Moreover, in mice with collagen-induced arthritis, HDL administration reduced IL-6 and TNF levels compared to untreated animals [43].

Unexpectedly, by testing the ability of HDL to reduce the secretion of inflammatory cytokines in macrophages insulted with LPS, no differences were observed between the CTR and RA groups, nor between the RA groups (remission/low-activity vs. active disease). Nonetheless, a positive correlation was observed between IL-6 secretion and the DAS28 score values in the individuals with active RA, indicating that the reduced anti-inflammatory activity of HDL relates to active disease. It is important to consider that HDL’s impairment in cholesterol efflux, as demonstrated in this study, may impact the inflammatory response. The intracellular accumulation of cholesterol, as well as the consequent increase in oxidative stress and endoplasmic reticulum stress, triggers inflammatory pathways that may worsen the clinical progression of RA. These pathways include activating apoptotic pathways and the inflammasome system, which triggers cell death through pyroptosis, linking inflammatory rheumatologic complications to atherosclerosis. Therefore, it can be inferred that the impairment in HDL function to remove cholesterol may, in the long term, stimulate inflammation and even compromise its anti-inflammatory activity. The role of HDL isolated from RA subjects on endoplasmic reticulum (ER) stress and inflammasome activation should be investigated.

It is noteworthy that the removal of excess cholesterol, in addition to reducing intracellular sterol accumulation, limits the oxidative and inflammatory stress that accompanies the evolution of the atherosclerotic plaque.

Cholesterol efflux mediated by HDL reduces the content and signaling of toll-like receptor 4 in macrophages, leading to a decrease in the release of inflammatory cytokines [44]. Moreover, as demonstrated by binary logistic regression analysis, the removal of cellular cholesterol by HDL was found to increase the OR of RA progressing to an active clinical form. The results of the present investigation demonstrated that increased HDLc, TC/apoB ratio, and HDL content in TC, as well as PL and apoA-1 were associated with a decrease in the OR for active disease in subjects with RA by 6.9%, 68.6%, 8.8%, 2.7%, and 6.8%, respectively. A reduction in the percentage of cholesterol efflux mediated by HDL increases the OR of an individual with RA having active disease by 26.2%. In this context, one must consider a vicious cycle, where the loss of HDL functionality contributes to active RA, which, through increased inflammatory insult, exacerbates the reduction in HDL functionality, thereby worsening the risk of atherosclerotic macrovascular disease.

The loss of HDL functionality may also help to better understand the lipid paradox described in subjects with RA presenting a high CV risk despite low plasma concentrations of TC and LDLc. Moreover, the inconsistencies regarding plasma lipids and CVD in RA may also be explained by the small number of individuals included in some restricted cohorts, as well as the considerable demographic and disease-related heterogeneity of the studies [17].

This study estimated the OR for the association between varying disease activity levels in RA and the plasma concentrations of HDLc, HDL particle composition, and functionality. However, since cross-sectional studies cannot establish causality, future longitudinal studies must explore cause-and-effect relationships. Based on the inclusion criteria of the study, the remission/low-activity group had a small number of participants included, which may represent a limitation of the present investigation. Considering the small sample size, a multivariate analysis was not performed. Nevertheless, the R-squared and power calculated achieved adequate values.

Many studies have addressed the effects of DMARDs, biological therapies, and Janus kinase inhibitors on plasma lipid profiles. These medications promote the reduction of inflammation, while at the same time, elevations in both plasma LDLc and HDLc levels are achieved [45,46,47,48,49,50,51]. Most of the RA subjects included in the present investigation were already being treated with these drugs before recruitment, which might explain the higher HDLc levels in the RA group in comparison to the CTR group.

The findings of this study suggest that the HDL composition and function may influence CVD risk in RA by affecting the disease’s inflammatory profile. These results should be validated through larger, long-term clinical trials to better establish the temporal relationship between HDL dysfunction and RA activity.

## 4. Materials and Methods

This cross-sectional cohort study comprised a group of individuals with RA and another group of control individuals. For the RA group, 70 individuals with RA treated at the Rheumatology Outpatient Clinic of the Hospital do Servidor Público Municipal de São Paulo (HSPM) were selected. A pre-selection was made based on the analysis of the rheumatology outpatient records of people with RA, and individuals who met the following criteria were chosen: age 18 years or older; both sexes; diagnosed with RA according to the 2010 ACR/EULAR classification criteria for RA (American College of Rheumatology/European League Against Rheumatism) [52]; and the absence of diabetes mellitus, pregnancy, liver failure, uncontrolled hypothyroidism, renal failure (estimated glomerular filtration rate < 60 mL/min/1.73 m^2^), nephrotic syndrome, body mass index (BMI) ≥ 40 kg/m^2^, other connective tissue diseases, smoking, and alcoholism. These pre-selected individuals were invited to participate in the study by telephone contact. Seventy healthy control individuals were selected from the local community, matched by age, sex, and BMI with the RA group and were invited to participate by telephone contact. In the control group, the exclusion criteria were the presence of any autoimmune disease, diabetes mellitus, pregnancy, liver failure, uncontrolled hypothyroidism, renal failure (estimated glomerular filtration rate < 60 mL/min/1.73 m^2^), nephrotic syndrome, BMI ≥ 40 kg/m^2^, smoking, and alcoholism. After excluding volunteers who did not meet the eligibility criteria and those who refused to participate in the study, 50 individuals were included in the control group and 56 in the RA group.

Height and weight were measured for BMI calculation, and obesity was defined as BMI ≥ 30 kg/m^2^. WC was determined with a measuring tape positioned halfway between the iliac crest and the lower rib margin, with values of >88 cm for women and >102 cm for men considered abdominal obesity. Information was collected regarding demographic data (age and sex) and physical activity (considering any intensity of regular physical activity and quantified in minutes per week). In the RA group, the use of non-steroidal anti-inflammatory drugs and the use and dose of prednisone and DMARDs were verified; in female participants, menopausal status, the use of hormone replacement therapy, and the use of hormonal contraceptives were questioned.

### 4.1. Evaluation of Inflammatory Activity in Individuals with RA

In participants with RA, on the day of blood sample collection, the DAS28 was used to measure the degree of disease activity, reflecting the degree of inflammation present. This score, DAS28, is a continuous composite index, which has been well-documented and validated, produced by the assessment of inflammatory markers such as the erythrocyte sedimentation rate (ESR), the individual’s global health assessment, and the medical examination of 28 joints to assess inflammation, pain, or normality in these joints. The following cutoff values for the DAS28 were considered: disease remission: <2.6; low-activity disease: 2.6 to <3.2; moderate-activity disease: 3.2 to 5.1; and high-activity disease: >5.1. The RA group was classified into 2 groups according to the parameters below: individuals with active disease, with DAS28-ESR ≥ 3.2, comprising those with moderate and high-activity disease; individuals in remission and with low-activity disease, with DAS28-ESR < 3.2.

### 4.2. Blood Collection and Plasma Lipid Determination

Peripheral venous blood was collected from all participants after an overnight fast of 8 h. Plasma and serum were immediately separated by centrifugation at 4 °C for 20 min at 3000 rpm. To obtain the plasma fraction containing only HDL, lipoproteins containing apoB (VLDL and LDL) were precipitated by adding a solution of dextran sulfate/magnesium chloride (1:1) to the plasma (100 μL/mL). Total plasma samples and plasma samples containing only HDL were kept at −80 °C.

Plasma lipids [ TC, HDLc, and TG) and lipids in the isolated HDL fraction (TC, TG, and PL) were determined by colorimetric enzymatic methods (Roche do Brasil, Sao Paulo, SP, Brazil). The concentration of apoA-1 in HDL and apoB in plasma was determined by immunoturbidimetry (Randox Brasil Ltd.a., São Paulo, SP, Brazil). The plasma concentration of LDL cholesterol (LDLc) was determined using the Friedewald formula [53], and non-HDL cholesterol (non-HDLc) was calculated by subtracting HDLc from TC. Atherogenic indices were calculated as the ratios of TG to HDLc and TC to apoB.

### 4.3. Isolation of Lipoproteins

The plasma density was adjusted with potassium bromide to 1.21 g/mL. LDL (d = 1.019–1.063 g/mL) and HDL (d = 1.063–1.21 g/mL) were isolated from plasma by ultracentrifugation in a discontinuous density gradient at 40,000 g for 24 h at 4 °C [54]. After isolation, the samples were dialyzed against phosphate buffer solution (PBS), and only the HDL was stored in a sucrose solution at −80 °C.

### 4.4. Acetylation of LDL

LDL was acetylated as previously described by Basu et al. [55] by adding a saturated solution of sodium acetate to LDL dissolved in 0.15 M sodium chloride and, subsequently, multiple small quantities of acetic anhydride. After intensive dialysis against PBS with EDTA (pH = 7.4), the samples were sterilized through a 0.22 µm filter, and the protein concentration was determined by the method of Lowry et al. [56].

### 4.5. Obtaining Bone Marrow-Derived Macrophages from Mice

Animal experiments were approved by the Ethics Committee on Animal Use (CEUA) of Nove de Julho University (# 6647311023; December 2023). The experimental protocols complied with the Ethical Principles of Animal Experimentation adopted by the Brazilian Society of Science in Laboratory Animals (SBCAL). C57Bl/6 mice were housed in the animal facility at 22 ± 2 °C, under a 12 h light/dark cycle, with free access to food and water. Animals were euthanized by intraperitoneal injection of an overdose of ketamine hydrochloride (300 mg/kg body weight) and xylazine hydrochloride (30 mg/kg body weight), following the guidelines of the National Council for Control of Animal Experimentation (CONCEA) of the Ministry of Science, Technology, and Innovation (MCTI). Bone marrow-derived cells were isolated from 6-week-old male mice, and macrophages were differentiated. Femurs and tibias were cleaned and isolated at the knee joint level. A 20 mL syringe with a 26-gauge needle containing bone marrow culture medium (DMEM with low glucose with 10% fetal bovine serum, 0.8% penicillin/streptomycin, and 10% L929 cell culture medium) was used to cut the end of each bone and to flush out the bone marrow from both bone ends. Bone marrow was aspirated and flushed using a 20 mL syringe with an 18 ½-gauge needle. The cells were centrifuged (1000 rpm at room temperature for 6 min), resuspended in culture medium, plated, and incubated for 5 days at 37 °C with 5% CO_2_. Then, the culture medium was changed to DMEM with low glucose containing 10% fetal bovine serum, 100 U/mL penicillin, and 100 µg/mL streptomycin [57].

### 4.6. Determination of HDL Anti-Inflammatory Activity in Macrophages

Macrophages were overloaded with acetylated LDL (50 µg/mL DMEM) and treated for 24 h with HDL (50 µg/mL DMEM) from individuals with RA or CTR. After washing, the cells were challenged with LPS (1 µg/mL) for 24 h. Control incubations were performed without HDL, followed by exposure to LPS. The culture medium was collected, and the amount of TNF and IL-6 was determined by ELISA (R&D Diagnostics, Minneapolis, MN, USA) [57].

### 4.7. Determination of Cholesterol Removal from Macrophages by HDL

Macrophages were incubated with DMEM (Low Glucose, Gibco, Grand Island, New York, USA) containing 1 mg of fatty acid-free albumin (FAFA) (Sigma-Aldrich, Steinheim, Germany), in the presence of 50 µg of acetylated LDL/mL and 0.3 µCi of ^14^C-cholesterol/mL (Amersham Biosciences, Buckinghamshire, United Kingdom) for 24 h. After washing with PBS containing FAFA, the cells were maintained in DMEM/FAFA to ensure the equilibrium of the intracellular pool of radioactive cholesterol for 18 h. Subsequently, they were washed with PBS/FAFA and incubated for 6 h with HDL (50 µg/mL) isolated from the control and RA groups. The culture medium was removed from the plates and centrifuged at 1500 rpm for 10 min at 4 °C to remove cellular debris, then it was transferred to scintillation vials, with the addition of counting solution (Perkin Elmer, Turku, Finland), and the radioactivity was determined in a beta counter (Beckman Coulter, Brea, CA, USA). The plate wells were washed twice with PBS, and the cellular lipids were extracted with hexane–isopropanol solution (3:2) (Merck, Darmstadt, Germany). After solvent evaporation, the remaining radioactivity in the cells was determined. The cells were solubilized in 250 µL of 0.2 N NaOH for 3 h at room temperature, and protein determination was performed. The percentage of cholesterol efflux was calculated as ^14^C-cholesterol in the medium/^14^C-cholesterol in the medium + ^14^C-cholesterol in the cells × 100. HDL-mediated specific efflux was calculated by subtracting the total efflux from that obtained with incubations with DMEM/FAFA only (basal efflux) [57].

### 4.8. Statistical Analysis

The Shapiro–Wilk test was used to assess the normality of quantitative variables. Parametric data are presented as the sample mean and standard deviation (SD), and when comparing two groups, Student’s *t*-test was used, with or without Welch’s correction, depending on the performance of the Levene test regarding the sphericity of the sample. When evaluating more than two samples, analysis of variance of non-repeated measures with Bonferroni post-test was employed. Non-parametric data are presented as the median and interquartile ranges (25% and 75%), and a comparison between the two groups was performed using the Mann–Whitney test. If normalization was necessary, the variables were transformed. The frequencies of categorical variables were compared using Fisher’s exact test, and associations between variables were analyzed using Pearson’s correlation. The univariate logistic regression was performed to evaluate the relationship between the clinical and laboratory variables and the disease activity (active or remission/low-activity RA). The logistic regression used each independent variable isolated to calculate the odds ratio and the 95% confidence interval. The Cox and Snell R-squared values were used to express each variable’s importance in the model. All independent variables used had the same size, and no transformation was applied. Although we did not perform a multivariate analysis, no variables presented a perfect multicollinearity with other variables studied. A significance level of *p* < 0.05 was considered statistically significant. The power was calculated using the G*Power software version 3.1, Düsseldorf, Germany [58]. Microsoft^®^ Excel for Mac 2023 and IBM^®^ SPSS Statistics for Mac (version 29 released in 2023 by IBM Corp., Armonk, NY, USA) software were used for data tabulation and analysis, respectively.

## Figures and Tables

**Table 1:** Age, anthropometric, and clinical data of CTR and RA groups.

	CTR *n* = 50	RA *n* = 56	*p*
Age (years)	56.5 (49.8–65.3)	62.5 (54.5–6.0)	0.116
Female	46 (92.0)	52 (92.9)	1.000
BMI (Kg/m^2^)	28.2 (25.9–31.3)	28.6 (25.4–31.7)	0.651
WC (cm)	96.0 (91.0–102.5)	96.0 (89.3–106.0)	0.761
Obesity	16 (32.0)	21 (37.5)	0.684
Dyslipidemia	22 (44.0)	18 (32.1)	0.233
Statin use	10 (20.0)	10 (17.9)	0.808
Post-menopausal	34 (73.9)	45 (86.5)	0.132
HRT	5 (10.9)	10 (19.2)	0.277
HC	3 (6.5)	0 (0.0)	0.100
Physical activity ≥ 150 min/wk	10 (20.0)	10 (17.9)	0.808
DAS28	4.15 (3.28–5.03)		
Disease remission	7 (12.5)		
Low-activity disease	6 (10.7)		
Moderate-activity disease	31 (55.4)		
High-activity disease	12 (21.4)		

RA: rheumatoid arthritis group; CTR: control group; BMI: body mass index; DAS28: Disease Activity Score with 28-joint counts; WC: waist circumference; HRT: hormonal replacement therapy; HC: hormonal contraceptive. Results are expressed as median and interquartile intervals (25% and 75%) or *n* (%). Data were compared using the Mann–Whitney test and Fisher’s exact test for categorical variables, always as two-sided analyses.

**Table 2:** Medications used in the RA group.

Drug	*n* (%)
Prednisone	8 (14.3)
Prednisone dose ≥ 7.5 mg/day	2 (3.6)
Conventional synthetic DMARDs	48 (85.7)
Hydroxychloroquine	10 (17.9)
Leflunomide	15 (26.8)
Methotrexate	40 (71.4)
Biological DMARDs	21 (37.5)
Anti-TNF	16 (28.6)
Abatacept	1 (1.8)
Tocilizumab	2 (3.6)
Rituximab	2 (3.6)
Target-specific synthetic DMARDs	2 (3.6)
Tofacitinib	2 (3.6)
Total	53 (94.6)

RA: rheumatoid arthritis group; DMARDs: disease-modifying antirheumatic drugs. The data are expressed as *n* (%).

**Table 3:** Plasma lipid profile and HDL particle composition of CTR and RA groups.

	CTR *n* = 50	RA *n* = 56	*p*
TC (mg/dL)	174 (147–195)	194 (171–206)	0.007
HDLc (mg/dL)	47 (38–54)	55 (43–62)	0.015
LDLc (mg/dL)	103 (83–128)	117 (98–127)	0.125
apoB (mg/dL)	35 (28–40)	35 (30–40)	0.874
non-HDLc (mg/dL)	122 (103–145)	139 (118–148)	0.630
TG (mg/dL)	87 (64–119)	96 (71–122)	0.362
TG/HDLc	1.8 (1.2–3.1)	1.8 (1.2–2.4)	0.735
TC/apoB	5.0 (4.6–5.7)	5.5 (5.0–6.0)	0.002
HDL particle composition			
HDL-TC (mg/dL)	42 (32–53)	46 (39–54)	0.216
HDL-TG (mg/dL)	8 (6–10)	8 (6–11)	0.401
HDL-PL (mg/dL)	84 (67–111)	103 (83–116)	0.002
HDL-apoA-1 (mg/dL)	44 (34–54)	53 (42–60)	0.009

RA: rheumatoid arthritis group; CTR: control group; TC: total cholesterol; HDLc: HDL cholesterol; LDLc: LDL cholesterol; TG: triglyceride; apoB: apolipoprotein B; HDL-TC: total cholesterol in HDL; HDL-TG: triglycerides in HDL; HDL-PL: phospholipids in HDL; HDL-apoA-1: apolipoprotein A-1 in HDL. Results are expressed as median and interquartile intervals (25% and 75%). Data were compared using the Mann–Whitney test, always as two-sided analyses.

**Table 4:** Age, anthropometric, and clinical data of RA groups according to the disease activity.

	Remission/Low-Activity Disease DAS28 < 3.2 (*n* = 13)	Active Disease DAS28 ≥ 3.2 (*n* = 43)	*p*
Age (years)	61 (42.5–63.0)	63 (56.0–68.0)	0.105
Female	11 (84.6)	41 (95.3)	0.227
BMI (Kg/m^2^)	26.5 (23.6–28.8)	29.2 (26.3–33.4)	0.032
WC (cm)	91 (89.5–97.5)	97 (89.0–108.0)	0.072
Obesity	2 (15.4)	19 (44.2)	0.101
Dyslipidemia	4 (30.8)	14 (32.6)	1.000
Statin use	1 (7.7)	9 (20.9)	0.424
Post-menopausal	9 (81.8)	36 (87.8)	0.630
HRT	0	10 (24.4)	0.096
HC	0	0	-
Physical activity ≥ 150 min/wk	3 (23.1)	7 (16.3)	0.682

RA: rheumatoid arthritis group; BMI: body mass index; DAS28: Disease Activity Score with 28-joint counts; WC: waist circumference; HRT: hormonal replacement therapy; HC: hormonal contraceptive. Results are expressed as median and interquartile intervals (25% and 75%) or *n* (%). Data were compared using the Mann–Whitney test and Fisher’s exact test for categorical variables, always as two-sided analyses.

**Table 5:** Plasma lipid profile and HDL particle composition of RA groups according to the disease activity.

	Remission/Low-Activity Disease DAS28 < 3.2 (*n* = 13)	Active Disease DAS28 ≥ 3.2 (*n* = 43)	*p*
TC (mg/dL)	192 (177–211)	194 (169–204)	0.719
HDLc (mg/dL)	60 (51–75)	50 (43–60)	0.021
LDLc (mg/dL)	109 (100–132)	122 (96–126)	0.662
apoB (mg/dL)	34 (28–37)	35 (30–41)	0.196
non-HDLc (mg/dL)	121 (115–146)	140 (120–149)	0.221
TG (mg/dL)	80 (56–88)	104 (77–135)	0.007
TG/HDLc	1.1 (0.9–1.7)	1.9 (1.5–3.2)	0.003
TC/apoB	5.8 (5.4–6.7)	5.3 (4.9–5.8)	0.014
HDL particle composition			
HDL-TC (mg/dL)	54 (47–68)	44 (34–51)	0.005
HDL-TG (mg/dL)	7 (6–12)	9 (6–11)	0.696
HDL-PL (mg/dL)	108 (90–128)	99 (79–116)	0.114
HDL-apoA-1 (mg/dL)	61 (47–69)	49 (39–58)	0.014

RA: rheumatoid arthritis group; TC: total cholesterol; HDLc: HDL cholesterol; LDLc: LDL cholesterol; TG: triglyceride; apoB: apolipoprotein B; HDL-TC: total cholesterol in HDL; HDL-TG: triglycerides in HDL; HDL-PL: phospholipids in HDL; HDL-apoA-1: apolipoprotein A-1 in HDL. Results are expressed as median and interquartile intervals (25% and 75%). Data were compared using the Mann–Whitney test, always as two-sided analyses.

**Table 6:** Binary logistic regression model for the RA group with active disease.

Active Disease (DAS28 ≥ 3.2) *n* = 43				
	Cox–Snell R^2^	OR	CI 95%	*p*
BMI (Kg/m^2^)	0.078	1.169	1.004–1.360	0.044
HDLc (mg/dL)	0.137	0.931	0.882–0.984	0.011
TG (mg/dL)	0.137	1.031	1.005–1.057	0.020
TC/apoB	0.139	0.314	0.126–0.781	0.013
HDL-TC (mg/dL)	0.181	0.912	0.853–0.976	0.007
HDL-PL (mg/dL)	0.074	0.973	0.947–1.000	0.048
HDL-apoA-1 (mg/dL)	0.127	0.932	0.882–0.985	0.013
HDL-mediated cholesterol efflux	0.165	0.738	0.591–0.921	0.007

DAS28: Disease Activity Score with 28-joint counts; BMI: body mass index; TC: total cholesterol; HDLc: HDL cholesterol; apoB: apolipoprotein B; HDL-TC: total cholesterol in HDL; HDL-PL: phospholipids in HDL; HDL-apoA-1: apolipoprotein A-1 in HDL; OR: odds ratio; CI: confidence interval.

## Data Availability

All data reported are included in the manuscript, and raw data can be kindly shared upon personal request to the corresponding author MP (m.passarelli@fm.usp.br or m.passarelli@uni9.pro.br).
